# Supplementary material for: Characterization of Beta-Lactamase and Fluoroquinolone Resistance Determinants in Escherichia coli, Klebsiella pneumoniae, and Pseudomonas aeruginosa Isolates from a Tertiary Hospital in Yola, Nigeria
Source: Trop Med Infect Dis. 2023 Nov 16;8(11):500. doi: 10.3390/tropicalmed8110500 (PMC10675496; doi:10.3390/tropicalmed8110500)
Supplement: Supplementary file 1 [file tropicalmed-08-00500-s001.zip › tropicalmed-2667367-supplementary.pdf]

Tropical Medicine and Infectious Disease

MDPI

Supplementary Materials: Characterization of Beta-Lactamase and Fluoroquinolone Resistance Determinants in *Escherichia coli*, *Klebsiella pneumoniae*, and *Pseudomonas aeruginosa* Isolates from a Tertiary Hospital in Yola, Nigeria

Diane E. Kawa [1,*], Isabella A. Tickler [2], Fred C. Tenover [3] and Shuwaram A. Shettima [4]

Table S1. Beta-lactam and fluoroquinolone resistance phenotypes in *E. coli, K. pneumoniae* and *P. aeruginosa* isolates collected during Period 1. MLST, multi-locus sequence type; ESBL, extended-spectrum beta-lactamase; AST, antimicrobial susceptibility testing; MEM, meropenem; ETP, ertapenem; IPM, imipenem.

ID	Organism	MLST	AST Phenotype			
			ESBL	Carbapenemase	Carbapenem Non-Susceptible	Fluoroquinolone Resistant
15949	*Escherichia coli*	ST692	None	metallo-beta-lactamase	MEM/ETP/IPM	Yes
16020	*Escherichia coli*	ST692	None	metallo-beta-lactamase	MEM/ETP/IPM	Yes
16029	*Escherichia coli*	ST692	None	metallo-beta-lactamase	MEM/ETP/IPM	Yes
16032	*Escherichia coli*	ST692	None	metallo-beta-lactamase	MEM/ETP/IPM	Yes
16047	*Klebsiella pneumoniae*	ST147	Yes	None	MEM/ETP	Yes
15958	*Pseudomonas aeruginosa*	ST2935	N/A	None	No	No
15964	*Pseudomonas aeruginosa*	ST1203	N/A	metallo-beta-lactamase	MEM/IPM	Yes
15965	*Pseudomonas aeruginosa*	ST773	N/A	metallo-beta-lactamase	MEM/IPM	Yes
15966	*Pseudomonas aeruginosa*	ST1203	N/A	metallo-beta-lactamase	MEM	Yes
15986	*Pseudomonas aeruginosa*	ST654	N/A	metallo-beta-lactamase	MEM/IPM	Yes
16014	*Pseudomonas aeruginosa*	ST244	N/A	None	No	No
16018	*Pseudomonas aeruginosa*	ST654	N/A	metallo-beta-lactamase	MEM/IPM	Yes
16048	*Pseudomonas aeruginosa*	ST1555	N/A	None	No	No

Table S2. Beta-lactam and fluoroquinolone resistant phenotypes in *E. coli, K. pneumoniae* and *P. aeruginosa* isolates collected during Period 2. MLST, multi-locus sequence type; ESBL, extended-spectrum beta-lactamase; AST, antimicrobial susceptibility testing; MEM, meropenem; ETP, ertapenem; IPM, imipenem.

ID	Organism	MLST	AST Phenotype			
			ESBL	Carbapenemase	Carbapenem Non-Susceptible	Fluoroquinolone Resistant
**17757**	***Escherichia coli***	Ambiguous (ST506, ST566)	ESBL	No	No	Yes
17758	*Escherichia coli*	Ambiguous (ST27, ST129)	No	No	No	No
17762	*Escherichia coli*	Ambiguous (ST27, ST129)	No	No	No	No
17771	*Escherichia coli*	ST2	No	No	No	No
17772	*Escherichia coli*	ST2	ESBL	No	No	Yes
17773	*Escherichia coli*	ST83	No	No	No	No
17775	*Escherichia coli*	Inconclusive (ST721, ST662, ST472)	ESBL	No	No	Yes
17776	*Escherichia coli*	Inconclusive (ST466, ST210, ST132)	No	No	No	Yes
17781	*Escherichia coli*	ST692	ESBL	serine-beta-lactamase	ETP	Yes
17782	*Escherichia coli*	ST471	ESBL	No	No	Yes
17786	*Escherichia coli*	ST132	No	No	No	Yes
17789	*Escherichia coli*	Inconclusive (ST500, ST437)	No	No	No	No
17792	*Escherichia coli*	ST2632	ESBL	No	No	Yes
17795	*Escherichia coli*	ST86	ESBL	No	No	Yes
17796	*Escherichia coli*	Ambiguous (ST566, ST506)	ESBL	No	No	Yes
17803	*Escherichia coli*	ST2	ESBL	No	No	No
17804	*Escherichia coli*	ST471	ESBL	No	No	Yes
17862	*Escherichia coli*	Inconclusive (ST500, ST437)	No	No	No	No
17864	*Escherichia coli*	ST58	ESBL	No	No	Yes
17759	*Klebsiella pneumoniae*	ST340	No	Metallo-beta-lactamase	MEM/ETP/IPM	Yes
17765	*Klebsiella pneumoniae*	ST86	No	No	No	No
17766	*Klebsiella pneumoniae*	ST20	No	No	No	No
17767	*Klebsiella pneumoniae*	ST392	No	No	No	Yes
17768	*Klebsiella pneumoniae*	ST340	No	Metallo-beta-lactamase	MEM/ETP/IPM	Yes
17770	*Klebsiella pneumoniae*	ST86	No	No	No	No
17785	*Klebsiella pneumoniae*	ST45	ESBL	No	No	No
17787	*Klebsiella pneumoniae*	ST2632	No	No	No	No
17790	*Klebsiella pneumoniae*	ST86	No	No	No	No
17793	*Klebsiella pneumoniae*	ST661	No	No	No	No
17861	*Klebsiella pneumoniae*	ST86	No	No	No	No
17863	*Klebsiella pneumoniae*	ST340	No	Metallo-beta-lactamase	MEM/ETP/IPM	Yes

17760	*Pseudomonas aeruginosa*	ST274	No	No	MEM/IPM	No
17761	*Pseudomonas aeruginosa*	Inconclusive (nearest ST244)	No	No	MEM/IPM	No
17763	*Pseudomonas aeruginosa*	ST773	No	Metallo-beta-lactamase	MEM/IPM	Yes
17777	*Pseudomonas aeruginosa*	ST773	No	Metallo-beta-lactamase	MEM/IPM	Yes
17778	*Pseudomonas aeruginosa*	ST773	No	Metallo-beta-lactamase	MEM/IPM	Yes
17779	*Pseudomonas aeruginosa*	ST773	No	Metallo-beta-lactamase	MEM/IMP	Yes
17791	*Pseudomonas aeruginosa*	ST773	No	Metallo-beta-lactamase	MEM/IMP	Yes
17794	*Pseudomonas aeruginosa*	ST773	No	Metallo-beta-lactamase	MEM/IMP	Yes
